# Role of Transferrin Receptor and the ABC Transporters ABCB6 and ABCB7 for Resistance and Differentiation of Tumor Cells towards Artesunate

**DOI:** 10.1371/journal.pone.0000798

**Published:** 2007-08-29

**Authors:** Gerhard Kelter, Daniel Steinbach, Venkata Badireenath Konkimalla, Tsuyoshi Tahara, Shigeru Taketani, Heinz-Herbert Fiebig, Thomas Efferth

**Affiliations:** 1 Oncotest GmbH, Institute of Experimental Oncology, Freiburg, Germany; 2 Department for Pediatrics, University of Ulm, Ulm, Germany; 3 Pharmaceutical Biology (C015), German Cancer Research Center, Heidelberg, Germany; 4 Department of Biotechnology, Kyoto Institute of Technology, Kyoto, Japan; Ordway Research Institute, United States of America

## Abstract

The anti-malarial artesunate also exerts profound anti-cancer activity. The susceptibility of tumor cells to artesunate can be enhanced by ferrous iron. The transferrin receptor (TfR) is involved in iron uptake by internalization of transferrin and is over-expressed in rapidly growing tumors. The ATP-binding cassette (ABC) transporters ABCB6 and ABCB7 are also involved in iron homeostasis. To investigate whether these proteins play a role for sensitivity towards artesunate, Oncotest's 36 cell line panel was treated with artesunate or artesunate plus iron(II) glycine sulfate (Ferrosanol®). The majority of cell lines showed increased inhibition rates, for the combination of artesunate plus iron(II) glycine sulfate compared to artesunate alone. However, in 11 out of the 36 cell lines the combination treatment was not superior. Cell lines with high TfR expression significantly correlated with high degrees of modulation indicating that high TfR expressing tumor cells would be more efficiently inhibited by this combination treatment than low TfR expressing ones. Furthermore, we found a significant relationship between cellular response to artesunate and TfR expression in 55 cell lines of the National Cancer Institute (NCI), USA. A significant correlation was also found for *ABCB6*, but not for *ABCB7* in the NCI panel. Artesunate treatment of human CCRF-CEM leukemia and MCF7 breast cancer cells induced *ABCB6* expression but repressed *ABCB7* expression. Finally, artesunate inhibited proliferation and differentiation of mouse erythroleukemia (MEL) cells. Down-regulation of ABCB6 by antisense oligonucleotides inhibited differentiation of MEL cells indicating that artesunate and ABCB6 may cooperate. In conclusion, our results indicate that ferrous iron improves the activity of artesunate in some but not all tumor cell lines. Several factors involved in iron homeostasis such as TfR and ABCB6 may contribute to this effect.

## Introduction

Artemisinin is a sesquiterpene isolated from *Artemisia annua* L., which is used in traditional Chinese medicine for the treatment of fever and chills [1]. Artemisinin reveals profound activity against *Plasmodium falciparum* and *Plasmodium vivax* [2], [3]. Artesunate and artemether are semi-synthetic derivatives of artemisinin with improved pharmacological features [4]. In addition to their anti-malarial activity, artemisinin and its derivatives are also active against cancer cells [5]–[9]. In both cases, the activity of the drugs is associated with the presence of iron. Iron is present in large excess bound to hemoglobin in erythrocytes, where the *Plasmodia* parasites are located. The active moiety of artemisinin-like drugs is an endoperoxide bridge, whose reductive homolysis is promoted by iron(II)-heme leading to C4-centered alkylating radicals [10]. These radical molecules cause macromolecular damage by alkylating essential malarial proteins inducing cell death of parasites [11]–[13]. On the other hand, iron content is higher in tumor cells than in normal cells [14] making them more susceptible to artemisinins. We and others have shown that the susceptibility of tumor cells to artemisinins can further be enhanced by the addition of transferrin or ferrous iron [15], [16].

The role of artemisinins and iron for malaria treatment has been intensively investigated during the past years [17], whereas the role of iron for tumor treatment with artemisinins is far less understood. The iron-binding protein, transferrin, is internalized into cancer cells after binding to the transferrin receptor (TfR, CD71). This is a transmembrane glycoprotein involved in iron uptake by internalization of transferrin. TfR exerts growth regulatory functions and is over-expressed in rapidly growing tumors [18]. The expression of TfR is of prognostic significance for several tumor types [19]–[21]. Ferrous iron can either be bound to transferrin or to other proteins before uptake.

The ATP-binding cassette (ABC) transporters ABCB6 and ABCB7 are involved in iron homeostasis. They are located in the mitochondria and transport heme and protoporphyrins into these organelles [22]. Most ABC transporters are involved in the active transport of phospholipids, ions, peptides, steroids, polysaccharides, amino acids, bile acids, pharmaceutical drugs and other xenobiotic compounds [23].

In humans, 49 different ABC transporters have been identified, which are classified into seven sub-families (A–G) [24]. In healthy organs several ABC transporters protect against the harmful effects of xenobiotics taken up with food. A high expression can, therefore, be found in the gastrointestinal tract, liver, and kidney. A protective function is also given as components of the blood brain barrier and the blood placenta barrier.

ABC transporters have been intensively investigated in the past years. ABCB1 (P-glycoprotein, *MDR1*), ABCC1-C6 (MRP1-6) and ABCG2 (BCRP) confer resistance to cytostatic drugs of tumors and contribute to the failure of tumor chemotherapy [25]. The mutated gene product of ABCC7 (CFTR) is involved in the pathogenesis cystic fibrosis, and some other mutated ABC transporter genes contribute to a number of hereditary diseases and disorders [24]. The pathogenic function of the majority of ABC transporters still awaits elucidation. To gain insight into functional aspects, we have developed a low density microarray for ABC transporters [26], [27]. Applying this microarray, we identified ABCA2 and ABCA3 as further genes involved in tumor drug resistance [28], [29].

In the present investigation, we focused on TfR, ABCB6, and ABCB7. The aim of the present study was to address the question, whether these proteins are involved in the response of tumor cells to artesunate.

## Materials and Methods

### Drugs

Artesunate was obtained from Saokim Ltd. (Hanoi, Vietnam) and iron(II) glycine sulfate (Ferrosanol®) from Sanol (Munich, Germany).

### Cell Lines

CCRF-CEM were cultured in RPMI medium and MCF-7 and MEL cells in DMEM medium each supplemented with 10% fetal calf serum under standard conditions (37°C, 5% CO_2_). Cells were passaged twice weekly.

Twenty-four cell lines out of Oncotest's 36 cell line panel were established at Oncotest from patient-derived tumor xenografts passaged subcutaneously in nude mice [30]. The origin of the donor xenografts have been previously described [31]. The cell lines T24, Panc1 and 22RV1 were obtained from ATCC (Rockville, MD, USA), the cell line LnCAP from DSMZ (Braunschweig, Germany) and the other 8 cell lines were kindly provided by the National Cancer Institute (Bethesda, MA, USA).

The cultivation of 55 cell lines (leukemia, melanoma, non-small cell lung cancer, colon cancer, renal cancer, ovarian cancer, brain tumors, prostate cancer, and breast cancer) of the Developmental Therapeutics Program of the NCI has been previously described [32].

### Effect on cell growth

MEL cells were cultured with and without 1.0 µg/ml artesunate (2.6 µM) for 24 and 48 h. Cell numbers were determined by trypan blue exclusion.

### Effect on differentiation

MEL cells (1×10^6^ cells/ml) were cultures in the presence of 2% DMSO with or without 1.0 µg/ml artesunate (2.6 µM) for 28h. The content of heme was estimated by the conversion of heme to protoporphyrin with oxalic acid [33 Taketani et al., 2003].

### Sulforhodamine B assay

*D*rug sensitivity of the NCI cell lines has been determined by the sulforhodamine B assay [34 Rubinstein et al., 1990]. The 50% inhibition concentration (IC_50_) values for artesunate have been reported [7] and those of other anti-cancer drugs have been deposited in the database of the NCI database (http://dtp.nci.nih.gov).

### Propidium iodide (PI) assay

A modified propidium iodide assay [35] was used to assess the compound's activity in Oncotest's 36 cell line panel. Briefly, cells were harvested from exponential phase cultures by trypsinization, counted and plated in 96 well flat-bottomed micro-titer plates at a cell density depending on the cell line (4.000–10.000 cells/well). After a 24 h recovery period to allow the cells to adhere and resume exponential growth, 10 µl of culture medium (six control wells/plate) or of culture medium containing the test compounds were added to the cells. The compounds were applied in triplicates at five concentrations. Following four days of continuous drug exposure, medium or medium with test compound, including all dead cells suspended in the culture medium, was aspirated and replaced by 200 µl of an aqueous propidium iodide (PI) solution (7 µg/ml). To measure the amount of living cells, cells were permeabilized by freezing the plates, resulting in the death of all cells that had remained attached to the bottom of the well after the incubation period. After thawing of the plates, fluorescence was measured using the Cytofluor 4000 micro-plate reader (excitation 530 nm, emission 620 nm), providing a direct relationship to the total viable cell number.

### Cell line array

Arrays of cell lines from the Oncotest Human Cell Line Collection were assembled using a tissue arrayer (Beecher Instruments, Sun Prairie, Wisc., USA) as recently described [36]. Pellet biopsies (0.6 mm in diameter) were taken from embedded cells and arrayed in duplicate in a new recipient paraffin block. Four-micrometer sections of the resulting microarray block were cut and transferred onto glass slides using the paraffin-sectioning aid system (Instrumedics, Hackensack, NJ, USA) and further processed for immunohistochemical staining. The arrays were stained with the primary antibody 71C03 against TfR (Neomarkers, Fremont, CA, USA). Staining was analyzed by light microscopy and semi-quantitatively evaluated as described [36].

### Quantitative real-time PCR

Quantitative PCR was done as described [37]. The ABI Prism 7700 Sequence Detector and Pre-Developed Assay Reagents (Applied Biosystems, Weiterstadt, Germany) were used for the quantification of all genes. The expression of the ABC transporters was standardized for the expression of two housekeeping genes, β-2-microglobulin (B2M) and Abelson gene 1 (ABL1). The ABI Prism 7700 Sequence Detector and Pre-Developed Assay Reagents (Applied Biosystems, Weiterstadt, Germany) were used for the quantification of all genes. The assay IDs were: B2M: Hs00187842_m1; ABL1: Hs00245445_m1; ABCB6: Hs00180568_m1 and ABCB7: Hs00188776_m1.

### Western blotting

MEL cells (1×10^6^ cells/ml) were cultured in the presence of 2% DMSO. After 24, 48, 72, or 96 h the cells were collected and lysed. The cellular proteins were analyzed by SDS-PAGE and transferred onto PVDF membranes. Immunoblotting was performed with antibodies for ABCB6, ABCB7, and actin as the primary antibodies [33].

### Transfection of oligonucleotides

Phosphorothioate sense and antisense oligonucleotides corresponding to the mouse ABCB6 gene were transfected using DOTAP transfection reagent (Roche Molecular Biochemicals, Mannheim, Germany) as described [33]

### Statistics

*Fisher's exact test* was used to calculate significance values as a measure for the dependency of two variables. This test was implemented into the WinSTAT Program (Kalmia, Cambridge, MA, U.S.A.). The TfR expression in the Oncotest panel of tumor cell lines has been determined by a tissue micro-array technique adapted for the examination of cell line and immunohistochemistry [36]. The results have been validated by conventional immunohistochemistry and Western blotting [36]. The mRNA expression values for *TFR* (clone M11507, GC102405) determined by microarray analysis [38], [39] were selected from the NCI database (http://dtp.nci.nih.gov). The microarray-based expression levels of *ABCB6* and *ABCB7* have been validated by RT-PCR [40].

## Results

### Transferrin Receptor (TfR)

To investigate the role of TfR and iron for cellular sensitivity to artesunate, Oncotest's 36 cell line panel was used. The 50% inhibition concentrations (IC_50_) for artesunate are shown in **Table 1**. The IC_50_ values varied between 0.197 and 47.779 µg/ml (0.512 and 124.295 µM respectively). The mean IC_50_ value was 2.791 µg/ml (7.261 µM). Furthermore, we tested a combination of artesunate and iron(II) glycine sulfate (10 µg/ml; Ferrosanol®) (**Table 1**). Here, the mean IC_50_ value was 0.978 µg/ml (2.544 µM) with a range from 0.013 and 22.96 µg/ml (0.026 and 59.748 µM, respectively). The data in **Table 1** are arranged according to the IC_50_ values for artesunate. In the majority of cell lines, the combination of artesunate plus iron(II) glycine sulfate resulted increased inhibition rates. However, in 11 out of the 36 cell lines (31%; OVXF 1619L, CNXF, 498NL, MCF7, RXF 393NL, MEXF 514L, MEXF 394NL, LXFL 529L, H460, 22RV1, RXF1781L, OVXL 899L) the combination treatment was not superior to treatment with artesunate alone (degree of modulation <1.2). Furthermore, degrees of modulation (IC_50_ for artesunate divided by the IC_50_ for artesunate plus iron(II) glycine sulfate) did not correlate with IC_50_ values for artesunate, e.g. cell lines with high IC_50_ values for artesunate were not more modulated by the addition of iron(II) glycine sulfate than cell lines with low IC_50_ values for artesunate (**Table 1**).

**Table 1:** TfR expression and in vitro anti-tumor activity of artesunate alone and in combination with iron(II) glycine sulfate (Ferrosanol®, 10 µg/ml) in Oncotest's 36 cell line panel.

Tumor Type	Cell Line	ART IC50 (µM)	ART+Ferrosanol IC50 (µM)	Degree of Modulation	TfR* Cell Microarray
Ovarian Ca	OVXF 1619L	0.512	0.747	0.69	
Gastric Ca	GXF 251L	0.578	0.193	3	0
Breast Ca	MAXF 401NL	0.752	0.536	1.4	0
Ovarian Ca	OVCAR3	1.168	0.871	1.34	0.33
Brain tumor	CNXF 498NL	1.233	1.514	0.81	
Lung Ca	LXFL 1121L	1.233	0.166	7.41	
Colon Ca	HT29	1.381	1.061	1.3	0
Head & neck tumor	HNXF 536L	1.566	0.026	60.2	
Breast Ca	MCF7	1.683	29.672	0.06	1
Pancreas Ca	PANC1	1.733	1.017	1.7	
Renal Ca	RXF 393NL	1.831	5.315	0.34	0
Endometrium Ca	UXF 1138L	2.125	1.272	1.67	0.33
Melanoma	MEXF 514L	2.177	2.448	0.89	0
Melanoma	MEXF 394NL	2.979	3.468	0.86	
Melanoma	MEXF 520L	4.121	0.502	8.21	
Bladder Ca	T24	4.519	1.332	3.39	
Prostate Ca	PC3M	5.341	0.341	15.67	3
Renal Ca	RXF 944L	6.532	1.08	6.05	1.33
Lung Ca	LXFA 526L	10.356	5.702	1.82	0
Brain tumor	SF268	11.181	0.034	330.62	
Melanoma	MEXF 462NL	12.503	0.674	18.56	0
Prostate Ca	DU145	12.503	4.188	2.99	0
Lung Ca	LXFL 529L	22.31	35.609	0.63	0
Prostate Ca	LNCAP	22.458	0.476	47.17	1.2
Lung Ca	H460	23.809	51.465	0.46	0.5
Pleura tumor	PXF 1752L	26.015	8.226	3.16	
Prostate Ca	22RV1	27.412	35.362	0.78	
Lung Ca	LXFA 629L	27.596	15.767	1.75	0
Bladder Ca	BXF 1218L	28.244	0.601	47	
Colon Ca	HCT116	31.054	18.351	1.69	
Renal Ca	RXF 1781L	41.228	36.589	1.13	
Ovarian Ca	OVXL 899L	46.785	58.988	0.79	0.33
Renal Ca	RXF 486L	53.293	2.747	19.4	1.33
Lung Ca	LXFA 289L	69.787	53.421	1.31	0
Melanoma	MEXF 276L	71.748	4.121	17.41	0.33
Pancreas Ca	PAXF 1657L	124.295	59.748	2.08	

* TfR expression by a cell micro-array technique has been described [36].

Then, we correlated the expression of TfR with the degrees of modulation. We found that cell lines with high TfR expression significantly correlated with high degrees of modulation (**Table 2**) indicating that high TfR expressing tumor cells could be more efficiently inhibited by a combination of artesunate and iron(II) glycine sulfate than low TfR expressing ones. TfR expression did not correlate with IC_50_ values for artesunate alone or artesunate plus iron(II) glycine sulfate.

**Table 2:** Relationship between transferrin receptor (TfR) protein expression and degree of modulation to a combination therapy of artesunate plus iron(II) glycine sulfate (see Table I) in tumor cell lines of the Oncotest panel.

Protein	Cut-off	Degree of Modulation		Fisher's
		≤3	> 3	Exact Test
TfR	<0.5*	14	2	
	≥0.5	1	4	p = 0.011

* Relative expression. For details see [36].

Furthermore, we investigated the association of *TFR* for sensitivity or resistance to artesunate in 55 cell lines of the NCI drug screening panel. We found a significant relationship between cellular response to artesunate and *TFR* expression (**Table 3**) indicating that *TFR* might be a determinant of artesunate sensitivity in tumor cells.

**Table 3:** Relationship between transferrin receptor (*TFR*), *ABCB6*, and *ABCB7* mRNA expression and response to artesunate (ART) in tumor cell lines of the NCI drug screening panel.

Gene	Cut-off	Artesunate		Fisher's
		sensitive*	resistant	Exact Test
*TFR*	<36.15**	9	17	
	>36.15	19	10	p = 0.022
*ABCB6*	<28.05	18	9	
	>28.05	10	18	p = 0.021
*ABCB7*	<−0.0482	13	14	
	>−0.0482	15	13	not significant

* The median log_10_IC_50_ value for artesunate was used as a cut-off to separate tumor cell lines as being “sensitive” or “resistant”

** Relative mRNA expression as reported (http://dtp.nci.nih.gov)

### ABCB6 and ABCB7

Since ABCB6 and ABCB7 are involved in iron homeostasis of cells, we investigated a role of these two proteins for cellular response to artesunate. Again, we used the IC_50_ values for artesunate of 55 NCI cell lines. These data were correlated with the microarray-based expression of *ABCB6* (clone AF070598, GC56272) and *ABCB7* (clone AA056272, GC10226). As shown in **Table 3**, a significant correlation was found for *ABCB6*, but not for *ABCB7*.

**Figure 1:**
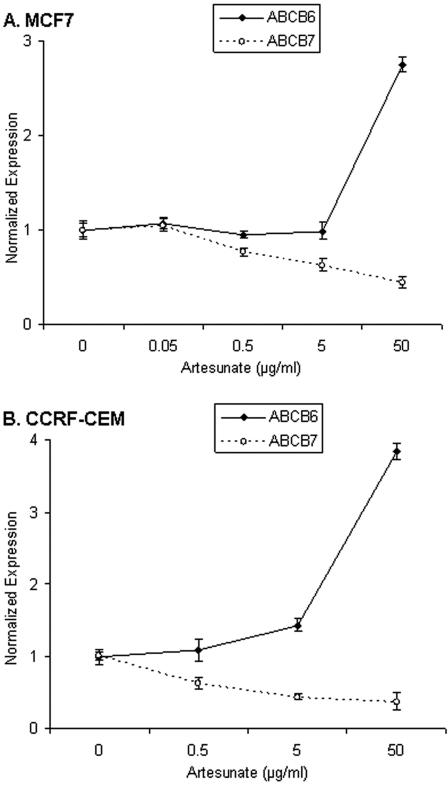
Expression of ABCB6 and ABCB7 in MCF7 (A.) and CCRF-CEM cells (B.) after treatment with artesunate. The expression after 24 hours is given in relation to the expression before treatment.

Then, MCF-7 breast cancer cells and CCRF-CEM leukemia cells were incubated with artesunate in a range of 0.05 to 50 µg/ml (0.13 to 130 µM respectively) for 24 h. Afterwards, mRNA was isolated and the expression of *ABCB6* and *ABCB7* determined by real time PCR. As can be seen in **Figure 1** the expression of *ABCB6* increased with increasing doses of artesunate, while the expression of *ABCB7* decreased accordingly. The induction of gene expression by artesunate might indicate a role of these two genes for cellular response to artesunate treatment. This can, however, not be taken as evidence, that the two genes are causatively related to artesunate response, and an epiphenomenal association can not be excluded.

**Figure 2:**
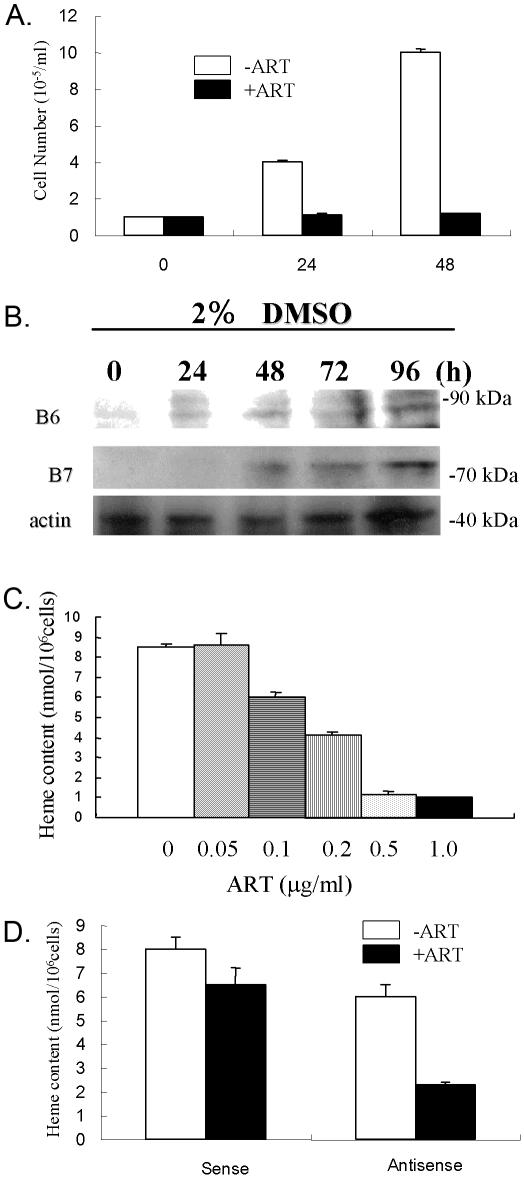
Effect of artesunate on MEL cell proliferation, differentiation, and ABCB6/ABCB7 expression. A. Effect on cell growth. MEL cells were cultured without or with 1.0 µg/ml artesunate for the indicated time. The cell numbers were measured by trypan blue exclusion. B. Protein expression of ABCB6 and ABCB7 after treatment with 2% DMSO for different time points. Actin expression served as loading control. C. Effect on differentiation. MEL cells (100,000 cells/ml) were cultured in the presence of various concentrations of artesunate for 48 h. The content of heme was estimated, by the method with oxalic acid. D. The cells transfected with sense- or antisense oligonucleotides for ABCB6 were induced with 2% DMSO without or with 0.18 µg/ml artesunate for 48 h.

Finally, we treated MEL cells with artesunate (1 µg/ml, 2.6 µM). Mouse erythroleukemia (MEL) cells were used as model to study the role of ABCB6 and ABCB7 during cellular differentiation. While cell growth was almost stopped, only 20% of cells died (**Figure 2A**). Treatment of MEL cells with 2% DMSO leads to erythroid differentiation and heme production (33). DMSO also induced the expression of ABCB6 and ABCB7 proteins (**Figure 2B**). Furthermore, the addition of artesunate completely blocked DMSO-induced erythroid differentiation in MEL cells in a dose-dependent manner (**Figure 2B**). Under these conditions most cells were alive. This indicates that artesunate is a potent inhibitor of proliferation and differentiation in MEL cells. Artesunate induced protein expression of both ABCB6 and ABCB7 (**Figure 2C**).

The IC_50_ value regarding heme production for artesunate in MEL cells was 0.18 µg/ml (0.468 µM). We previously reported that an antisense oligonucleotide directed against ABCB6 depressed the erythroid differentiation of MEL cells to 75% [33]. When MEL cells were transfected with this antisense oligonucleotide and treated with artesunate (0.18 µg/ml, 0.468 µM) in combination, the heme content decreased to about 30% control (**Figure 2D**). This indicates an additive effect.

## Discussion

The aim of the present study was to explore the role of proteins related to iron homeostasis for response of tumor cells to artesunate. For this purpose, we focused on TfR and the ABC transporters, ABCB6 and ABCB7.

We observed a wide heterogeneity of tumor cell lines towards artesunate. Whereas iron(II) glycine sulfate was able to increase sensitivity of tumor cells to artesunate in most cell lines, there was no correlation between the IC_50_ values and high degrees of modulation by iron(II) glycine sulfate. This indicates that iron(II) glycine sulfate is not a typical resistance modulator comparable to modulators of multidrug resistance, where modulating agents completely or partially overcome resistance of a drug-resistant sub-line to a cytostatic drug towards the level of a parental drug-sensitive cell line [25].

It is important to point out that several cell lines did not show increased inhibition by the combination of artesunate and iron(II) glycine sulfate compared to artesunate alone. Some of these cell lines did even reveal decreased efficacy to artesunate under co-treatment with iron(II) glycine sulfate. Iron is an important regulator of cell growth and tumor cells may take advantage of external iron supply to grow faster. Iron(II) glycine sulfate is an approved drug, Ferrosanol® applied for many years in the clinic to treat iron deficiency. Therefore, iron(II) glycine sulfate seems to be a safe and suitable adjunct for combination treatments with artesunate at first sight. Our results do, however, indicate that there may be patients, who would not profit from addition of iron(II) glycine sulfate during artesunate treatment. In this context, iron(II) glycine sulfate is a double edged sword improving treatment response in the majority of tumor cell lines but worsening it in others. Other iron preparations have also been reported to improve the response of tumor cells to artemisinins, e.g. ferrous sulfate or iron-loaded transferring (holotransferrin) [9], [15]. It remains to be analyzed, whether the same is true for these iron preparations.

The fact that in some cases iron(II) gylcine sulfate could also lead to reduced response of tumor cells towards artesunate opens an option for the testing of tumors before treatment in a clinical setting. If predictive tests, e.g., determination of TfR expression or sensitivity testing by MTT assay, indicate enhanced sensitivity, a combination therapy of artesunate and iron(II) glycine sulfate could be applied. If this is not the case, iron(II) glycine sulfate could be skipped, and artesunate could be applied alone. This situation adds to the attractiveness of individualized tumor therapy. The main idea of this concept is to adapt treatment protocols of each individual patient according to the results of pre-therapeutic testing of efficacy of a planned therapy [41].

On the other hand, three out of the five melanoma cell lines were clearly above-average sensitive to artesunate (individual IC_50_<4.2 µM), and both more resistant melanomas (IC_50_>12 µM) were strongly sensitized (degree of modulation >15) by the addition of iron(II) glycine sulfate indicating that this entity is especially suited for this treatment regimen. Interestingly, these results are supported by recent clinical data. Artesunate has been applied to two patients with otherwise drug-resistant and refractory uveal mealoma [42]. The prognosis of refractory uveal melanoma patients is generally worse (3–5 months). One artesunate treated patient with uveal melanoma had a stable disease following artesunate treated, but finally the tumor progressed and the patient died after 24 months. The second patients with uveal melanoma was treated with artesunate plus iron(II) glycine sulfate and is still living after more than 50 months [42]. This may be taken as a clue that the combination of artesunate plus iron(II)glycine sulfate is indeed especially suited for this tumor type. Further analyses have to prove this hypothesis.

Interestingly, the expression of *TFR* correlated with IC_50_ values for artesunate alone in the panel of 55 NCI cell lines, but not in the panel of 36 Oncotest cell lines. This is a clue that *TFR* expression is only one determinant of response of tumor cells towards artesunate. While endogenous iron might contribute to artesunate sensitivity, exogenous iron application during artesunate treatment is favorable for those tumors with high *TFR* expression levels.

In addition to TfR, iron is transported in cells by ABCB6 and ABCB7. Therefore, we also investigated the role of these two proteins for treatment of tumor cells with artesunate. ABCB6 is involved in the biosynthesis of heme via interaction with ferrochelatase, which is regulated by iron [33]. We found that the microarray-based mRNA expression of *ABCB6* but not of *ABCB7* correlated with IC_50_ values for artesunate in the NCI cell line panel. While *ABCB6* was induced upon artesunate treatment in MCF7 and CCRF-CEM cells, *ABCB7* expression was down-regulated. These results speak at least in the case of ABCB6 for a role in determining sensitivity to artesunate.

In addition to the anti-proliferative activity of artesunate on tumor cells, we also analyzed its effect on differentiation. For this reason, we used MEL cells, which are an established model to study ABCB6 and ABCB7 during cellular differentiation [43]. DMSO induced both differentiation and up-regulation of ABCB6 and ABCB7 protein expression. Artesunate was able to reverse DMSO-induced differentiation was measured by cellular heme production. Down-regulation of ABCB6 by transfection with antisense oligonucleotides inhibited DMSO-induced heme biosynthesis indicating that this protein might play a role in differentiation.

The role of ABCB6 and ABCB7 for drug resistance has been sparsely investigated as of yet. Boonstra et al. [44] did not find that ABCB6 expression in small lung cancer cells was associated with resistance to mitoxantrone. Thirteen different ABC transporters including ABCB6 and different anti-apoptotic Bcl2 gene family members were reported to be amplified in various drug-resistant cell lines [45]. While the other factors may contribute to clinical response towards neo-adjuvant chemotherapy, the specific role of ABCB6 remains unanswered in this study. Park et al. [46] analyzed the ABC transporter gene expression profiles in breast cancer patients who underwent neo-adjuvant chemotherapy. Several ABC transporters including ABCB6 showed significantly increase expression in residual disease. Again, the role of ABCB6 remains elusive. An association between ABCB7 and drug resistance has not been reported yet.

In conclusion, our results indicate that ferrous iron improves the activity of artesunate in some but not all tumor cell lines. Several factors involved in iron homeostasis such as TfR and ABCB6 may contribute to this effect. It remains to be analyzed, whether other factors of iron metabolism (ferrochelatase, ferritin etc) are also relevant for response of tumor cells towards artesunate
